# Outbreaks Associated with Treated Recreational Water — United States, 2000–2014

**DOI:** 10.15585/mmwr.mm6719a3

**Published:** 2018-05-18

**Authors:** Michele C. Hlavsa, Bryanna L. Cikesh, Virginia A. Roberts, Amy M. Kahler, Marissa Vigar, Elizabeth D. Hilborn, Timothy J. Wade, Dawn M. Roellig, Jennifer L. Murphy, Lihua Xiao, Kirsten M. Yates, Jasen M. Kunz, Matthew J. Arduino, Sujan C. Reddy, Kathleen E. Fullerton, Laura A. Cooley, Michael J. Beach, Vincent R. Hill, Jonathan S. Yoder

**Affiliations:** ^1^Division of Foodborne, Waterborne, and Environmental Diseases, National Center for Emerging and Zoonotic Infectious Diseases, CDC; ^2^Oak Ridge Institute for Science and Education, Oak Ridge, Tennessee; ^3^Environmental Protection Agency, Washington, D.C.; ^4^Division of Emergency and Environmental Health Services, National Center for Environmental Health; ^5^Division of Healthcare Quality Promotion, National Center for Emerging and Zoonotic Infectious Diseases, CDC; ^6^Division of Bacterial Diseases, National Center for Immunization and Respiratory Diseases, CDC.

Outbreaks associated with exposure to treated recreational water can be caused by pathogens or chemicals in venues such as pools, hot tubs/spas, and interactive water play venues (i.e., water playgrounds). During 2000–2014, public health officials from 46 states and Puerto Rico reported 493 outbreaks associated with treated recreational water. These outbreaks resulted in at least 27,219 cases and eight deaths. Among the 363 outbreaks with a confirmed infectious etiology, 212 (58%) were caused by *Cryptosporidium* (which causes predominantly gastrointestinal illness), 57 (16%) by *Legionella* (which causes Legionnaires’ disease, a severe pneumonia, and Pontiac fever, a milder illness with flu-like symptoms), and 47 (13%) by *Pseudomonas* (which causes folliculitis [“hot tub rash”] and otitis externa [“swimmers’ ear”]). Investigations of the 363 outbreaks identified 24,453 cases; 21,766 (89%) were caused by *Cryptosporidium*, 920 (4%) by *Pseudomonas*, and 624 (3%) by *Legionella*. At least six of the eight reported deaths occurred in persons affected by outbreaks caused by *Legionella*. Hotels were the leading setting, associated with 157 (32%) of the 493 outbreaks. Overall, the outbreaks had a bimodal temporal distribution: 275 (56%) outbreaks started during June–August and 46 (9%) in March. Assessment of trends in the annual counts of outbreaks caused by *Cryptosporidium, Legionella,* or *Pseudomonas* indicate mixed progress in preventing transmission. Pathogens able to evade chlorine inactivation have become leading outbreak etiologies. The consequent outbreak and case counts and mortality underscore the utility of CDC’s Model Aquatic Health Code (https://www.cdc.gov/mahc) to prevent outbreaks associated with treated recreational water.

An outbreak associated with recreational water is the occurrence of similar illnesses in two or more persons, epidemiologically linked by location and time of exposure to recreational water or to pathogens or chemicals aerosolized or volatilized from recreational water into the surrounding air. Public health officials in the 50 states, the District of Columbia, U.S. territories, and Freely Associated States* voluntarily report outbreaks associated with recreational water to CDC. This report focuses on data in two groups of outbreaks associated with treated recreational water: 1) those that started during 2000–2012 and were previously summarized (*1*) and 2) those that started during 2013–2014 and were electronically reported to the Waterborne Disease and Outbreak Surveillance System (WBDOSS)^†^ by December 31, 2015 (https://www.cdc.gov/healthywater/surveillance/rec-water-tables-figures.html). Data on each outbreak included case count,^§^ number of deaths, etiology, setting (e.g., hotel) and venue (e.g., pool, hot tub/spa) where the exposure occurred, and earliest illness onset date. Poisson regression analysis was conducted to assess the trend in the annual counts of outbreaks, except when overdispersion required the use of negative binomial regression analysis.

During 2000–2014, public health officials from 46 states and Puerto Rico reported 493 outbreaks associated with treated recreational water, which resulted in at least 27,219 cases (Table) and eight deaths. Etiology was confirmed for 385 (78%) outbreaks. Among these, 363 (94%) were caused by pathogens (including four caused by both *Cryptosporidium* and *Giardia*) and resulted in at least 24,453 cases. Twenty-two (6%) outbreaks were caused by chemicals and resulted in at least 1,028 cases. Among the 363 outbreaks with a confirmed infectious etiology, 212 (58%) were caused by *Cryptosporidium*, 57 (16%) by *Legionella*, and 47 (13%) by *Pseudomonas*. Of the 24,453 cases, 21,766 (89%) were caused by *Cryptosporidium*, 920 (4%) by *Pseudomonas*, and 624 (3%) by *Legionella*. Of the 212 outbreaks caused by *Cryptosporidium*, 24 (11%) each affected >100 persons; four of these outbreaks each affected ≥2,000 persons. At least six of the eight deaths,^¶^ which all occurred after 2004, were in persons affected by outbreaks caused by *Legionella*.

**TABLE:** Number of outbreaks associated with treated recreational water, total and median number of cases, by etiology — United States, 2000–2014

Etiology	No. (%) of outbreaks		No. (%) of cases	Median no. (range) of cases per outbreak
**Total**		**493 (100)**	**27,219 (100)**	**10 (2–5,697)**
**Bacterium**		**129 (26)**	**1,899 (7)**	**6 (2–119)**
*Bacillus*		1 (0)	20 (0)	20 (—*)
*Campylobacter*		2 (0)	10 (0)	5 (4–6)
*Escherichia coli*		6 (1)	86 (0)	12.5 (2–31)
*Legionella*		57 (12)	624 (2)	3 (2–107)
MRSA		1 (0)	10 (0)	10 (—)
Nontuberculous mycobacteria		2 (0)	14 (0)	7 (3–11)
*Pseudomonas*		47 (10)	920 (3)	10 (2–119)
*Salmonella*		1 (0)	5 (0)	5 (—)
*Shigella*		11 (2)	207 (1)	12 (3–56)
*Staphylococcus*		1 (0)	3 (0)	3 (—)
**Parasite**		**220 (45)**	**21,976 (81)**	**14 (2–5,697)**
*Cryptosporidium*		208 (42)	21,626 (79)	14.5 (2–5,697)
*Giardia*		8 (2)	210 (1)	8.5 (3–149)
*Cryptosporidium, Giardia*		4 (1)	140 (1)	37 (3–63)
**Virus**		**14 (3)**	**578 (2)**	**36 (6–140)**
Echovirus		1 (0)	36 (0)	36 (—)
Norovirus		13 (3)	542 (2)	36 (6–140)
**Chemical**		**22 (4)**	**1,028 (4)**	**17.5 (2–665)**
Excess chlorine, disinfection by-product, or altered pool chemistry		22 (4)	1028 (4)	17.5 (2–665)
**Unidentified**		**108 (22)**	**1,738 (6)**	**7.5 (2–280)**

**Abbreviation:** MRSA = methicillin-resistant *Staphylococcus aureus*.

* Not applicable because only one outbreak was nationally reported for that etiology.

Hotels** (i.e., hotels, motels, lodges, or inns) were the leading setting associated with 157 (32%) of the 493 outbreaks. Of the 157 hotel-related outbreaks, 94 (60%)^††^ had a confirmed infectious etiology, 40 (43%) were caused by *Pseudomonas*, 29 (31%) by *Legionella*, and 17 (18%) by *Cryptosporidium*.^§§^ Sixty-five (41%) hotel-related outbreaks were associated with hot tubs/spas, and 47 (30%) started during February–March. Among all 493 outbreaks, a bimodal temporal distribution was observed. The 275 (56%) outbreaks that started during June–August were predominantly caused by *Cryptosporidium,* whereas the 46 (9%) that started in March were predominantly caused by an unidentified etiology or pathogens other than *Cryptosporidium* (Figure 1). Negative binomial regression analysis indicated that during 2000–2007, the annual number of outbreaks caused by *Cryptosporidium* increased by an average of 25% (95% confidence interval [CI] = 7%–45%) per year (Figure 2). No significant trend was found after 2007.^¶¶^ Poisson regression analysis indicated that during 2000–2014 the annual number of outbreaks caused by *Legionella* increased by an average of 13% (95% CI = 6%–21%) per year, and the annual number of *Pseudomonas* folliculitis outbreaks (a total of 41 outbreaks during 2000–2014) decreased by an average of 22% (95% CI = 14%–29%) per year.***

**FIGURE 1:**
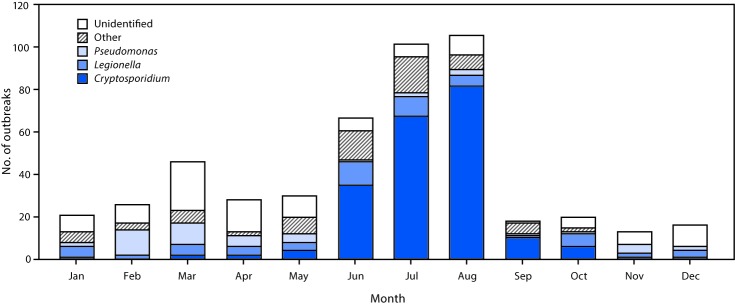
Number of outbreaks associated with treated recreational water (N = 493), by etiology and month — United States, 2000–2014* * Includes outbreaks with the following etiologies: *Bacillus, Campylobacter, Escherichia coli*, methicillin-resistant *Staphylococcus aureus*, nontuberculous mycobacteria, *Salmonella, Shigella, Staphylococcus, Giardia*, echovirus, norovirus, or excess chlorine/disinfection by-product/altered pool chemistry.

**FIGURE 2:**
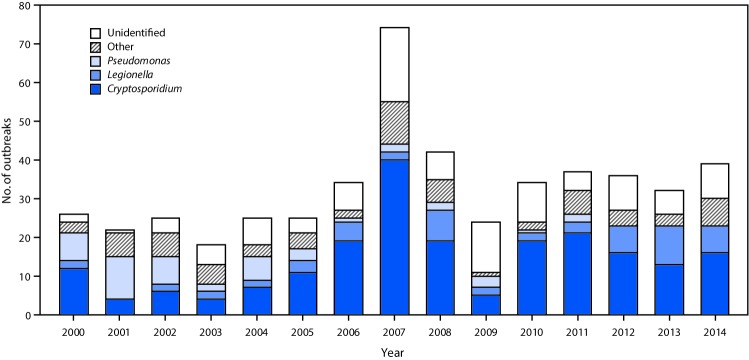
Number of outbreaks associated with treated recreational water (N = 493), by etiology and year — United States, 2000–2014 * Includes outbreaks with the following etiologies: *Bacillus, Campylobacter, Escherichia coli,* methicillin-resistant *Staphylococcus aureus*, nontuberculous mycobacteria, *Salmonella, Shigella, Staphylococcus, Giardia*, echovirus, norovirus, or excess chlorine/disinfection by-product/altered pool chemistry.

## Discussion

Approximately 500 outbreaks associated with treated recreational water occurred in the United States during 2000–2014. The most frequently reported outbreak setting was hotels. Approximately half of the outbreaks started during June–August, followed by a smaller peak in March. The second peak might reflect swimming’s transition from an only-summertime to a year-round activity, as the relative number of indoor versus outdoor treated recreational water venues increases. The aquatics sector and public health can voluntarily adopt CDC’s Model Aquatic Health Code to improve the design, construction, operation, and maintenance of public (nonbackyard) treated recreational water venues to prevent illness and injury.

Chlorine is the primary barrier to the transmission of pathogens in treated recreational water. At CDC-recommended concentrations of at least 1 ppm,^†††^ free available chlorine inactivates most pathogens within minutes although extremely chlorine-tolerant *Cryptosporidium* can survive for >7 days (*2*,*3*). *Cryptosporidium* is transmitted when a diarrheal incident (i.e., a high-risk *Cryptosporidium* contamination event) occurs in the water and the contaminated water is ingested. The parasite’s extreme chlorine tolerance enables it to persist in water, cause outbreaks that sicken thousands, and spread to multiple recreational water venues and other settings (e.g., child care settings). Rates of individual cases caused by *Cryptosporidium* peak in the summer, coinciding with the summer swim season (*4*).

In contrast, *Legionella* and *Pseudomonas* are effectively controlled by halogens (e.g., chlorine and bromine) in well-maintained treated venues. However, because these pathogens can persist in biofilm (where microbial cells inhabit a primarily polysaccharide matrix, the cells cannot be removed from a surface by gentle rinsing) (*5*), and they are protected from inactivation and amplify when disinfectant concentrations are not properly maintained. Approximately 20% of 13,864 routine inspections of public hot tubs/spas conducted in 16 jurisdictions in 2013 identified improper disinfectant concentrations (*6*). *Legionella* is typically transmitted when aerosolized water droplets (e.g., produced by hot tub/spa jets) containing this bacterium are inhaled, whereas *Pseudomonas* is transmitted when skin comes in contact with contaminated water. Multiple factors contribute to *Legionella* and *Pseudomonas* growth in hot tubs/spas, including inadequate disinfectant concentration; warm (77°F–108°F [25°C–42°C]) water temperatures (which facilitate pathogen amplification and make it difficult to maintain adequate disinfectant concentration); water aeration (which depletes halogens); and the presence of biofilm on wet venue surfaces, scale, and sediment (*7*). The increasing annual rate of Legionnaires’ disease cases (286% during 2000–2014) (*8*), and possibly, the significantly increasing annual number of outbreaks caused by *Legionella*, might be associated with increasing size of susceptible populations (persons aged ≥50 years or those with chronic disease [particularly chronic lung disease] or who are immunocompromised; current or former smokers; or cancer patients), and increased *Legionella* growth in the environment, as well as increased awareness of the disease with improved testing and reporting (*8*). The significantly decreasing number of annual *Pseudomonas* folliculitis outbreaks might reflect an actual decrease or possibly focusing on hot tub/spa remediation to prevent further transmission rather than outbreak investigation and reporting.

If a diarrheal incident occurs in treated recreational water or an outbreak at least suspected to be caused by *Cryptosporidium* occurs, CDC recommends hyperchlorination, i.e., chlorinating water to achieve 3-log_10_ (99.9%) *Cryptosporidium* inactivation^§§§^ (https://www.cdc.gov/healthywater/swimming/aquatics-professionals/fecalresponse.html). Alternatively, ultraviolet light or ozone systems can be added to inactivate *Cryptosporidium*, particularly in venues at increased risk for contamination (e.g., those intended for children aged <5 years, who might have limited or no toileting skills). As in any public setting, treated venues in the hotel setting should be operated and maintained by a trained operator or responsible supervisor.^¶¶¶^ These and other recommendations can be found in CDC’s Model Aquatic Health Code. CDC also provides specific recommendations for disinfecting hot tubs/spas contaminated with *Legionella* (https://www.cdc.gov/legionella/downloads/hot-tub-disinfection.pdf). Investigations of Legionnaires’ disease outbreaks indicate that effective water management programs for buildings and treated recreational water venues (e.g., hot tubs/spas) at increased risk for *Legionella* growth and transmission can reduce the risk for Legionnaires’ disease (*8*,*9*).

The findings in this report are subject to at least two limitations. First, the outbreak counts presented likely underestimate the actual incidence, in part because of variation in public health capacity and reporting requirements across jurisdictions. Second, reporting and review procedures (e.g., increased completeness of data on outbreaks caused by *Legionella*) changed over time, which affects the ability to compare data across years.

Addressing the challenges presented by chlorine-tolerant and biofilm-associated pathogens require sustained attention to improving design, construction, operation, and management of public treated recreational water venues. This includes educating the public. Preventing *Cryptosporidium* contamination is critical to preventing transmission. Thus, the key message to the public, particularly parents of young bathers, is “Don’t swim or let your kids swim if sick with diarrhea.” Preventing transmission of *Legionella*, *Pseudomonas*, and other chlorine-susceptible pathogens means educating bathers and parents of young bathers to check the inspection scores of public treated recreational water venues and conduct their own mini-inspection before getting into the water (e.g., measure bromine or free chlorine level and pH with test strips, which can be purchased at pool supply, hardware, and big-box stores). Potential hot tub/spa users should know whether they are at increased risk for Legionnaires’ disease, so they can choose to avoid hot tubs/spas, as indicated (https://www.cdc.gov/legionella/downloads/fs-legionnaires.pdf). The halting of the substantial increase in annual numbers of outbreaks caused by *Cryptosporidium* might, at least in part, be because of local, state, and federal Healthy and Safe Swimming Week (the week before Memorial Day) campaigns (*10*). Thus, the focus of these campaigns could regularly be expanded beyond preventing *Cryptosporidium* transmission in an effort to prevent other recreational water outbreaks.

**Summary**

**What is already known about this topic?**

Outbreaks associated with treated recreational water can be caused by pathogens or chemicals.

**What is added by this report?**

During 2000–2014, 493 outbreaks associated with treated recreational water caused at least 27,219 cases and eight deaths. Outbreaks caused by *Cryptosporidium* increased 25% per year during 2000–2006; however, no significant trend occurred after 2007. The number of outbreaks caused by *Legionella* increased 14% per year.

**What are the implications for public health practice?**

The aquatics sector, public health officials, bathers, and parents of young bathers can take steps to minimize risk for outbreaks. The halting of the increase in outbreaks caused by *Cryptosporidium* might be attributable to Healthy and Safe Swimming Week campaigns.

## Footnotes

* Includes Federated States of Micronesia, Marshall Islands, and Republic of Palau.

† 2013–2014 were the last years for which finalized data were available. For more information on WBDOSS, visit https://www.cdc.gov/healthywater/surveillance/index.html; outbreaks resulting from recreational water exposures on cruise ships are not reported to WBDOSS.

§ Based on the estimated number of primary cases. For outbreaks that started before 2009, if both the actual and estimated case counts were reported, the estimated case count was used if the population was sampled randomly or the estimated count was calculated by applying the attack rate to a standardized population.

¶ The two remaining deaths were in persons affected by an outbreak caused by an etiology that was unidentified but suspected to be *Legionella*.

** Other settings: community/municipality/public park (115 [23%] outbreaks), club/recreational facility (68 [14%]), waterpark (54 [11%]), private residence (31 [6%]), subdivision/neighborhood (21 [4%]), school/college/university (14 [3%]), unidentified (13 [3%]), camp/cabin setting (nine [2%]), child care/daycare center/day camp (six [1%]), health care facility (three [1%]), and other (two [0%]). Categories were not consistently used or defined over the study period.

†† Approximately half (60 [56%]) of the 108 outbreaks with an unidentified etiology were associated with the hotel setting. Among the 60 outbreaks, 23 (38%) started during March–April; 41 (68%) were outbreaks of skin-related illness.

§§ The remaining eight outbreaks were caused by norovirus (five [5%] outbreaks), *Bacillus* (one [1%]), nontuberculous mycobacterium (one [1%]), and *Staphylococcus* (one [1%]).

¶¶ The 2007 number of outbreaks associated with treated recreational water and caused by *Cryptosporidium* (40), and thus, of outbreaks overall might be outliers. For 2007, Utah reported a statewide outbreak primarily associated with treated recreational water and caused by *Cryptosporidium*; neighboring states reported additional outbreaks associated with recreational water and caused by *Cryptosporidium*. All of these individual outbreaks might have been a single multistate outbreak. https://www.cdc.gov/mmwr/preview/mmwrhtml/ss6012a1.htm.

*** Because of concerns that folliculitis might not be cultured, which means the etiology cannot be identified, a Poisson regression analysis was conducted to assess the annual number of outbreaks of skin-related illness caused by *Pseudomonas* or an unidentified etiology. The annual number of outbreaks decreased by 5% (95% CI = 1%–9%).

††† At water pH ≤7.5 and temperature ≥77°F (25°C).

§§§ At water pH ≤7.5 and temperature ≥77°F (25°C), 3-log_10_ *Cryptosporidium* inactivation can be achieved in the absence of cyanuric acid (which prevents chlorine degradation by the sun’s ultraviolet light but substantially delays pathogen inactivation) by maintaining free available chlorine at 20 ppm for 12.75 hours and in the presence of 1–15 ppm cyanuric acid by maintaining free available chlorine at 20 ppm for 28 hours.

¶¶¶ Trained operators are those who have successfully completed an approved operator training course; responsible supervisors are those who can conduct and record results of water quality testing, properly maintain water quality, perform general maintenance procedures, and identify when to close venues to protect public health.
